# Isothermal pumping analysis for high-altitude tethered balloons

**DOI:** 10.1098/rsos.140468

**Published:** 2015-06-17

**Authors:** Kirsty A. Kuo, Hugh E. M. Hunt

**Affiliations:** Department of Engineering, University of Cambridge, Trumpington Street, Cambridge CB2 1PZ, UK

**Keywords:** climate engineering, tethered balloons, sulfur dioxide, titanium dioxide

## Abstract

High-altitude tethered balloons have potential applications in communications, surveillance, meteorological observations and climate engineering. To maintain balloon buoyancy, power fuel cells and perturb atmospheric conditions, fluids could be pumped from ground level to altitude using the tether as a hose. This paper examines the pumping requirements of such a delivery system. Cases considered include delivery of hydrogen, sulfur dioxide (SO_2_) and powders as fluid-based slurries. Isothermal analysis is used to determine the variation of pressures and velocities along the pipe length. Results show that transport of small quantities of hydrogen to power fuel cells and maintain balloon buoyancy can be achieved at pressures and temperatures that are tolerable in terms of both the pipe strength and the current state of pumping technologies. To avoid solidification, transport of SO_2_ would require elevated temperatures that cannot be tolerated by the strength fibres in the pipe. While the use of particle-based slurries rather than SO_2_ for climate engineering can reduce the pipe size significantly, the pumping pressures are close to the maximum bursting pressure of the pipe.

## 1. Introduction

High-altitude tethered balloons are an emerging technology with potential applications in the fields of communications, meteorological measurements, surveillance, power harvesting and climate engineering. There are many engineering issues associated with the design of such a system [1], and this paper addresses one significant challenge: the pumping requirements for transporting materials along the length of the tether. Using the tether as a hose would provide a cost- and energy-efficient means of: (i) delivering lighter-than-air gases to the balloon, to maintain internal balloon pressures for long-term deployment; (ii) delivering materials that can be used to power fuel cells, thereby avoiding wires along the length of the tether; and (iii) delivering materials such as reflective particles that could be used to perturb atmospheric conditions and alter global temperatures. The latter forms the engineering concept of the SPICE (Stratospheric Particle Injection for Climate Engineering) Project, under which this work on pumping considerations was carried out. Alternative proposed climate engineering delivery systems include high-altitude aircraft, missiles, rigid towers and single-use balloons [2,3]. The use of a tether as a feed line for replenishment of lifting gases is not novel, having been described previously [4].

The basic engineering design of the system considered here is as follows: the balloon is flying in the stratosphere, at a height of about 20 km, probably in a region where relatively low wind speeds can be expected for a significant part of the year. The balloon tether serves both to fix the balloon and to contain a pipe through which the material is to be pumped. In this study, which constitutes an initial design assessment, only a single balloon at maximum altitude and a single, continuous tether will be considered. Further lift could be supplied by using multiple balloons or lifting devices attached along the tether, and further pumping capacity could be provided by using multiple tethers attached to a single balloon.

There are many engineering challenges associated with this system, but for the purposes of this paper it is assumed that the tether is formed from a pipe of circular cross-section that is wrapped with high strength fibres to resist the bursting pressures, which are in turn surrounded by axial fibres to carry the tension forces. This fibre arrangement is discussed by Causier [5]. The bursting pressures in the tether are largest at ground level, as this is where the greatest fluid pressure exists, and decrease with altitude, whereas the tension forces are smallest at ground level, and increase along the length of the tether to a maximum at the connection point to the balloon.

The tether fibres need to have high strength, low weight and to be electrically non-conducting to reduce the risk of lightning strikes. Causier [5] discusses candidate fibres and identifies Kevlar 49 and poly-*p*-phenylenebenzobisoxazole (PBO) as two options. As with many fibres, these materials will creep, and if the stresses are high enough they will creep to failure. As these processes are thermally activated, it will be taken as axiomatic that it is desirable to keep temperatures at or below ambient temperatures at ground level. Causier [5] analyses the stresses in a tether with separate hoop and axial fibres and shows that the maximum bursting pressure of the pipe depends on the ratio of the transverse and axial modulus of the fibres. The maximum bursting pressures are 139 MPa for Kevlar 49, and 130 MPa for PBO [5]. These values could be increased if the fibres are embedded in resin or if additional fibres are added between layers, and contain some uncertainty due to the limited data available on the transverse fibre modulus [5]. However, as a conservative estimate, these values will be used in this paper as the maximum allowable pressures in the pipe.

This paper presents an initial isothermal pumping analysis of such a tether system. Beginning with a number of representative materials for transportation, the implications for pumping pressures, temperatures and pipe design are discussed. The limitations of the isothermal assumption are discussed in §5.

## 2. Material selection

The representative materials considered here are hydrogen (H_2_), sulfur dioxide (SO_2_) and a mixture of titanium dioxide particles and nitrogen (TiO_2_/N_2_).

### 2.1 Hydrogen

Hydrogen is the lightest existing gas, making it an excellent candidate for use as a lifting gas in the balloon. While safety precautions will be required to manage the risk of explosion due to the flammability of hydrogen, it is nevertheless proposed as the preferred lifting gas over the inert and heavier helium due to its abundance and low manufacturing cost. The small size of the hydrogen molecule will cause diffusion through the balloon skin over time leading to a loss in pressure. By pumping hydrogen through the tether, the buoyancy of the balloon can be controlled for both long-term deployment and management of the balloon launch and recovery procedure. Hydrogen can also be used to run fuel cells, providing a clean, reliable and lightweight source of power at altitude.

The required mass flow rate of hydrogen is estimated based on a 10% loss of balloon gas volume per week, and the operation of a 3 kW fuel cell. The magnitude of the balloon gas loss is dependent on factors such as the resistance of the balloon skin material to gas diffusion and the integrity of the balloon skin. The assumed value of 10% loss per week can be considered a mid-range estimate, based on the experience of a high-altitude balloon specialist (Andy Elson 2011, personal communication). A balloon of 100 m radius, which will provide lift of 343 tonnes at 20 km, will require 0.69 m^3^ s^−1^ of hydrogen to replace the lost gas, which equates to 4.9×10^−3^ kg s^−1^ of hydrogen at ambient atmospheric conditions of −60°C and 6.3 kPa (absolute—equivalent to 800 Pa gauge pressure). A 3 kW fuel cell with 25% efficiency and calorific value of 39 kWh kg^−1^ will require a flow rate of 8.5×10^−5^ kg s^−1^ at an inlet pressure of 1 MPa. Combining these requirements, we will calculate the pumping requirements for a system that delivers 5.0 × 10^−3^ kg s^−1^ of hydrogen at 1 MPa to 20 km altitude. This value is relatively insensitive to the power output of the fuel cell, as the flow rate required by the fuel cell is small compared with the balloon gas replenishment. To estimate the sensitivity of the results due to the chosen parameters, calculations are also performed for a 30% loss of balloon gas volume per week, and a 50 m radius balloon.

### 2.2 Sulfur dioxide

SO_2_ is chosen as a candidate material due to its demonstrated cooling effect on global temperatures. The 1991 Mount Pinatubo event injected around 20 million tonnes of SO_2_ and hydrogen sulfide (H_2_S) into the stratosphere, resulting in an associated peak annual mean forcing of −2.97 W m^−2^ [6], and a global mean temperature reduction of about 0.5°C for a period of about 2 years [7]. Climate engineering proposals seek to mimic this effect by spraying SO_2_ aerosol particles into the stratosphere. SO_2_ is readily available, non-flammable and not as toxic as H_2_S, although significant safety measures would be required. There are a number of possible side effects of SO_2_ injection [8,9].

The quantity of materials to be pumped over 1 year for climate engineering is taken as the amount of material required to achieve the same dimming effect as the Pinatubo eruption: 13.64 Mt of SO_2_ [10]. This amount will increase the Earth's Bond albedo by 0.015. An estimated Bond albedo increase of about 0.018 is required to counteract the effects of a doubling of atmospheric carbon dioxide [11]. Simulation suggests one Pinatubo-size injection of SO_2_ per year will produce a steady-state global mean temperature change of 5°C [6]. It will be assumed that this quantity of materials will be delivered by four tethered-balloon systems, each operating for 300 days per year. This gives a required mass flow rate of 132 kg s^−1^ of SO_2_ per pipe. To estimate the sensitivity of the results due to the chosen parameters, calculations are also performed for delivering the same total amount of material using 8 and 40 balloons, and maximum (exit) flow velocities of 10 and 50 m s^−1^.

### 2.3 Titanium dioxide particles in nitrogen

Pope *et al.* [10] propose TiO_2_ particles with an effective radius of 0.11 μm as an alternative cooling agent to SO_2_. These particles give a magnified dimming effect compared to SO_2_ aerosols due to increased light scattering and are already produced in large quantities at the submicrometre size. Other advantages of using TiO_2_ over SO_2_ include scope for relatively small-scale field experiments to study particle properties (equivalent studies for SO_2_ would require significantly longer timescales and larger test areas), and potentially using surface coatings to minimize ozone depletion reactions, but much research needs to be carried out to confirm this [10]. For these reasons, TiO_2_ will be considered as a candidate particle, although many of the arguments used can be transferred to other particles (including alumina, silica, silicon carbide, etc.) if they are preferred. The pumping of TiO_2_ particles will require a carrier fluid. Using nitrogen as a carrier is considered here, as the introduction of nitrogen into the stratosphere will have no damaging environmental effect, poses no dangers to human health and is readily and cheaply available. H_2_O is dismissed as a carrier fluid as, apart from the large pumping pressures that would be required due to the liquid density, the introduction of moisture into the dry environment of the stratosphere would have negative impacts of stratospheric chemistry [8].

The pumping of mixtures of gas and solids and liquids and solids is well established, and widely used in the agricultural, oil, mining and food industries. These industries often need to pump solids over long distances, both horizontally and vertically. For conveying particles in gas over long distances with high velocities, a dilute phase system is used. Typically, this involves conveying the material in suspension in the flowing gas, with a phase density *ϕ* (ratio of mass flow rate of solids to mass flow rate of gas) of less than 10, and usually in the range of 0.3–0.9 (Bill Layton 2012, personal communication). The primary effect of adding particles to the fluid is an increased head pressure, and the particles are so fine that they are considered to have no effect on the frictional flow losses. A phase density ratio of 0.5 is used in this paper. To keep the material in suspension, a minimum value of conveying line velocity of 13–15 m s^−1^ is recommended [12]. To achieve an equivalent dimming effect as the Pinatubo eruption, 10.3 Mt of TiO_2_ is required per year [10]. This gives a required mass flow rate of 298 kg s^−1^ TiO_2_/N_2_ (phase density 0.5: 99.3 kg s^−1^ TiO_2_, 199 kg s^−1^ N_2_) for each of the four tethered-balloon systems operating for 300 days per year. To investigate the sensitivity of the results to the chosen parameters, calculations are also performed for delivering the same total amount of material using phase densities of 0.3 and 0.9, 8 and 40 balloons, and maximum (exit) flow velocities of 10 and 50 m s^−1^.

## 3. Thermodynamic properties

The thermodynamic properties for the candidate materials are obtained from the NIST Reference Fluid Thermodynamic and Transport Properties Database (REFPROP) [13].

### 3.1 Hydrogen

**Figure 1.**
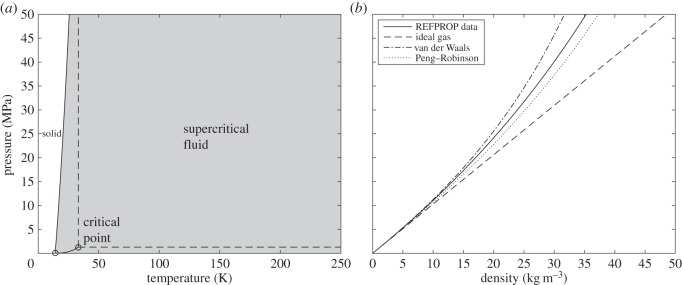
(*a*) Phase diagram for H_2_. (*b*) Pressure versus density for H_2_ at 250 K.

The phase diagram for H_2_ is shown in figure 1*a*. The REFPROP database contains thermodynamic properties for the shaded region, which extends to 1000 K and 2000 MPa. These properties are calculated using an equation of state from Leachman *et al*. [14], and the solid–liquid line is constructed from data given in [15]. Figure 1*b* shows the variation of density with pressure for H_2_ at 250 K. The uncertainty in the REFPROP data for density is approximately 0.04% [13]. To capture the variation of density with pressure and temperature, a suitable equation of state is required, and three commonly used equations of state are included in this figure: the ideal gas law; van der Waals equation; and the Peng–Robinson equation. The ideal gas law is based on the assumptions of negligible molecular size and intermolecular attractions, and as such is unsuitable for modelling gases at high densities and high pressures. For H_2_ at 250 K and up to 50 MPa, the Peng–Robinson equation provides a good model of real gas behaviour.

### 3.2 Sulfur dioxide

**Figure 2.**
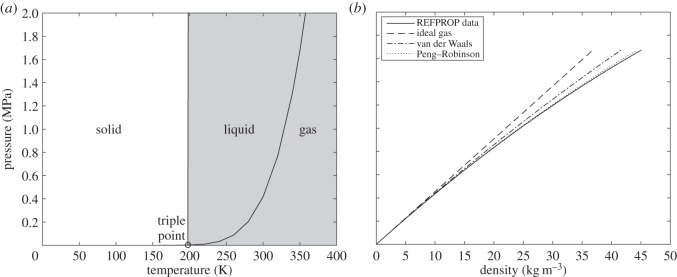
(*a*) Phase diagram for SO_2_. (*b*) Pressure versus density for SO_2_ at 350 K.

The phase diagram for SO_2_ is shown in figure 2*a*. The dark shaded region indicates the region of available data from the REFPROP, which is calculated based on equations of state from Lemmon & Span [16] and Ihmels *et al.* [17]. The data used to construct the solid–liquid line are from Hogenboom *et al.* [18]. At room temperature, the freezing of SO_2_ occurs at 300 MPa [19,20]. However, apart from this value, there is little data available on the pressures and temperatures at which freezing of SO_2_ occurs, which will be shown below to be critical. At 250 K, the density is largely invariant with pressure, as is expected for a liquid, and is approximately 1530 kg m^−3^. Figure 2*b* shows the variation of density with pressure for SO_2_ at 350 K (gas phase). The uncertainty in the REFPROP data for density is approximately 0.5% [13]. For SO_2_ at 350 K and pressures up to condensation at 1.67 MPa, the Peng–Robinson equation provides a good model of real gas behaviour.

### 3.3 Nitrogen

**Figure 3.**
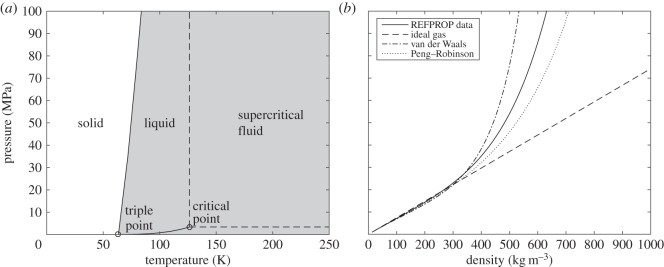
(*a*) Phase diagram for N_2_. (*b*) Pressure versus density for N_2_ at 250 K.

The properties of nitrogen at extremes of pressure and temperature are well documented, and extrapolation of data is not necessary for this case. The phase diagram for N_2_ is shown in figure 3*a*, constructed using data from Span *et al.* [21]. At room temperature and pressure, N_2_ exists as a gas and is considered a supercritical fluid when the pressure exceeds the critical pressure of 3.396 MPa. Figure 3*b* shows the variation of density with pressure for N_2_ at 250 K, as given in REFPROP and calculated using various equations of state. The uncertainty in the REFPROP data for density is approximately 0.02% [13]. For nitrogen at pressures less than 20 MPa, the ideal gas equation of state provides a reasonably accurate model of the thermodynamic properties. At higher pressures, the Peng–Robinson equation provides the closest-fitting model.

## 4. Pumping analysis

This section presents a pumping analysis for the three fluids based on the assumption of isothermal flow through a constant-radius pipe. The assumption of constant-temperature conditions has been chosen as it provides a convenient means of simplifying the pumping analysis. Section 5 presents a discussion of the limitations of this assumption.

For isothermal flow through a vertical pipe of constant radius *r*, the change in fluid pressure *P* with height *z* is the sum of three contributions: the frictional pressure loss, the static head and the change in momentum, respectively, expressed as

4.1 $$-\frac{\text{d}P}{\text{d}z}=f\frac{\rho V^{2}}{4r}+\rho^{*}g+\frac{\text{d}(\rho^{*}V^{2})}{\text{d}z},$$

where *f* is the Darcy Friction Factor, *ρ* is the fluid density, *V* is the conveying line velocity and ρ* is the slurry density for the TiO_2_/N_2_ case given as

4.2 $$\rho^{*}=\frac{\rho_{s}\rho_{f}(\phi +1)}{\phi \rho_{f}+\rho_{s}},$$

where *ρ*_*s*_ and *ρ*_*f*_ are the densities of solid (TiO_2_) and fluid (N_2_), respectively. When only fluids are being pumped, ρ* is equivalent to *ρ*. The value of the Darcy Friction Factor is conservatively assumed to be 0.02 for Reynolds numbers above 10^5^.

The Peng–Robinson equation of state provides a relationship between the fluid pressure and density

4.3 $$P=\frac{\rho RT}{M-\rho b}-\frac{\rho^{2}a\alpha }{M^{2}-2bM\rho -\rho^{2}b^{2}},$$

where *R* is the universal gas constant, *T* is the fluid temperature, *M* is the molar mass and *a*, *b*, *α* are parameters that are functions of the fluid's critical properties, acentric factor and temperature. Substituting equations (4.2) and (4.3) into equation (4.1) gives an expression for the change in density with height, which can be solved numerically. By defining the fluid outlet conditions at 20 km altitude, the variation of pressure, density and velocity along the length of the pipe can be determined.

The required pipe internal radius is found using the condition of constant mass flow:

4.4 $$\dot{m}=\rho^{*}\pi r^{2}V.$$

and the defined fluid outlet conditions. This equation also provides the relationship between fluid density and velocity along the pipe.

### 4.1 Hydrogen

**Figure 4.**
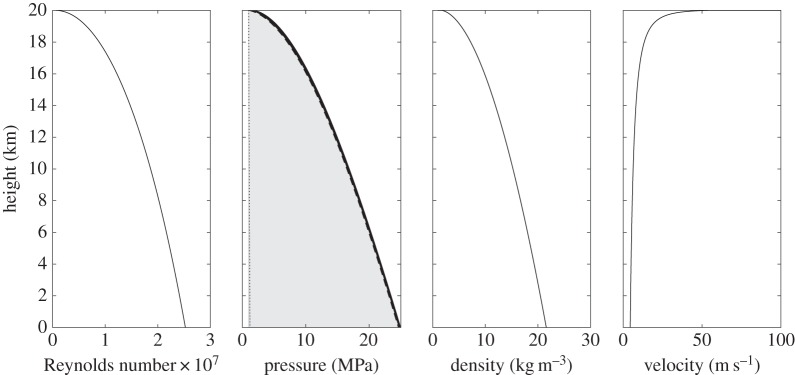
Variation of fluid properties for pumping H_2_ at 250 K. The pressure contribution from the static head is indicated by the dotted line, the contribution from change in fluid momentum is negligible, and the solid line shows the total pressure requirement, which is dominated by the frictional pressure loss.

It is assumed that the pumping of hydrogen will occur above 33.145 K, the critical temperature above which the hydrogen exists as a gas or supercritical fluid. To avoid creep in the pipe's strength fibres, the pumping temperature should be kept below ambient at ground level. For this isothermal analysis, a pumping temperature of 250 K is used. The pressure and density of the hydrogen will vary from high values at ground level to lower values at 20 km. H_2_ will expand as it travels up the pipe, and if the pipe diameter is kept constant the flow velocity will increase to a maximum at the outlet. Setting the maximum fluid velocity to 100 m s^−1^ and the outlet pressure to 1 MPa, in accordance with the fuel cell specification, the required radius to give a mass flow rate of 4.97×10^−3^ kg s^−1^ is 4.05×10^−3^ m. The resulting variation of fluid properties with height is shown in figure 4. The Reynolds number assumes a constant dynamic viscosity of 8.00 μPa s. The required pumping pressure at ground level is 24.6 MPa, and the travel time for the fluid is 50 min.

To investigate the sensitivity of the results to changes in balloon size, gas loss rate and flow velocity, calculations are also performed for a 50 m radius balloon, a 30% loss of balloon gas per week, and maximum (exit) flow velocities of 10 m s^−1^ and 50 m s^−1^. The results are shown in tables 1 and 2. It can be seen from these values that, perhaps counterintuitively, the required pumping pressure decreases as the amount of fluid to be pumped increases (due to a larger balloon size, or greater gas loss rate). This is because the pumping pressure is dominated by the frictional pressure loss contribution, which is inversely proportional to the pipe radius. A doubling of the exit velocity results in a smaller pipe and more-than-double the required pumping pressure. The travel time generally decreases with the amount of fluid to be pumped but is relatively invariant to changes in exit velocity. This latter effect can be attributed to the shape of the velocity curve in figure 4: the flow velocity shows only a relatively slow increase along the first 19 km of pipe, with the maximum acceleration occurring in the final 1 km section. This means that increasing the exit velocity reduces the travel time in the uppermost section of pipe but has little effect elsewhere.

**Table 1.** Required pumping pressures for 10% loss of balloon gas per week.

balloon radius (m)	50			100		
balloon volume (m^3^)	5.24×10^5^			4.19×10^6^		
lift (tonnes)	42.8			343		
mass flow rate H_2_ (kg s^−1^)	6.95×10^−4^			4.97×10^−3^		
exit velocity (m s^−1^)	10	50	100	10	50	100
pipe radius (m)	4.79×10^−3^	2.14×10^−3^	1.52×10^−3^	1.28×10^−2^	5.73×10^−3^	4.05×10^−3^
pumping pressure (MPa)	2.58	17.6	42.6	1.80	10.4	24.6
travel time (minutes)	62	75	82	47	44	50

**Table 2.** Required pumping pressures for 30% loss of balloon gas per week.

balloon radius (m)	50			100		
balloon volume (m^3^)	5.24×10^5^			4.19×10^6^		
lift (tonnes)	42.8			343		
mass flow rate H_2_ (kg s^−1^)	1.92×10^−3^			1.47×10^−2^		
exit velocity (m s^−1^)	10	50	100	10	50	100
pipe radius (m)	7.96×10^−3^	3.56×10^−3^	2.52×10^−3^	2.21×10^−2^	9.87×10^−3^	6.98×10^−3^
pumping pressure (MPa)	2.12	13.3	32.2	1.68	7.85	18.7
travel time (minutes)	53	57	63	43	34	38

The required pumping pressure for these combinations of balloon size, gas loss rate and velocity fall safely within the fibre strength limits, and the pipe radius values are small, which is advantageous in minimizing tether drag. This indicates that, within the range of parameter values considered here in this isothermal analysis, the pumping of hydrogen for powering fuel cells and maintaining balloon pressure is technically feasible.

### 4.2 Sulfur dioxide

The density of liquid SO_2_ is approximately 1530 kg m^−3^ at 250 K. If SO_2_ is to be pumped to 20 km in the liquid phase, the pressure at the base must overcome a static head of approximately 310 MPa, and the additional frictional pressure losses, say a total of 400 MPa. This is well above 300 MPa, the pressure at which SO_2_ transforms into the solid phase at room temperature, and this exceeds the pipe's maximum bursting pressure, hence pumping liquid SO_2_ from ground level is not feasible. The required pressure could be reduced by using in-line pumps but this would add significantly to the complexity of the system so will not be considered further here.

**Figure 5.**
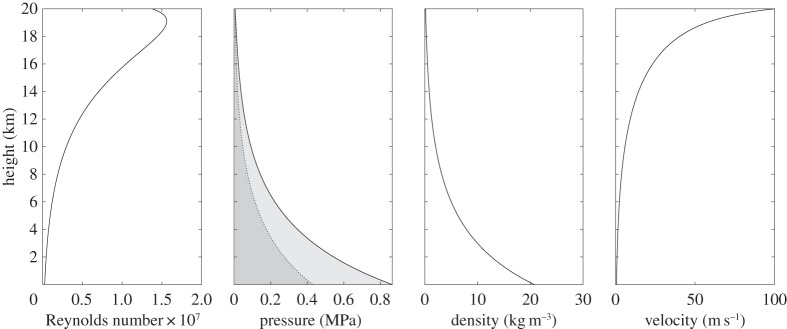
Variation of fluid properties for pumping SO_2_ at 350 K. The pressure contribution from the static head is indicated by the dotted line and the dark shaded region; the contribution from frictional pressure loss is shown by the light shaded region and the change in momentum is negligible. The solid line shows the total pressure requirement.

The pressure requirements for pumping SO_2_ can be reduced by using SO_2_ in a lower density state, that is, at an elevated temperature where the SO_2_ exists as a gas or supercritical fluid. This will require careful management of the flow temperatures and pressures, to ensure (i) that the fluid does not approach the higher density liquid region, as this would result in a rapid escalation in the required pumping pressures and (ii) that the pipe's fibres are not heated to temperatures that induce rapid creep failure. The latter condition is extremely problematic, as the long-term operation of a pumping system at elevated temperatures will provide a continual heat input to the pipe, establishing a temperature gradient between the internal and external surfaces and leading to variable creep rates through the pipe thickness. For the purpose of this analysis, it will be assumed that the pipe can be sufficiently insulated and a constant fluid temperature of 350 K will be used. At the top of the pipe, the SO_2_ particles will evaporate and no additional pressure is required for dispersion. Setting the maximum fluid velocity to 100 m s^−1^ and the outlet pressure to 5.5 kPa (atmospheric pressure at 20 km), the required radius to give a mass flow rate of 132 kg s^−1^ is 1.86 m. The resulting variation of fluid properties with height is shown in figure 5. The Reynolds number assumes a constant dynamic viscosity of 21.6 μPa s, and the required pumping pressure at ground level is 865 kPa.

Although the required pumping pressure at ground level is relatively small and therefore achievable, the pipe radius is large, resulting in an extremely heavy system and large drag forces due to wind effects. The total fluid mass carried within the pipe is 942 tonnes, which (ignoring the mass of the pipe, balloon, payload and other important considerations) by itself would require a 280 m diameter hydrogen-filled balloon for lift in zero wind conditions. To reduce the pipe weight and drag, a variable pipe radius could be used. By varying the pipe radius from 1.86 m at 20 km to a smaller value at ground level, the fluid will be forced to occupy a smaller volume, resulting in higher pressures, densities and velocities. This could be achieved by stepping the pipe radius, although this would require joints that can effectively withstand high tensile forces and high pressures. Instead, it is proposed to continually vary the pipe radius with length. Engineering the pipe radius in this way could ensure that the flow velocities and required pumping pressures remain below a feasible value, and the size of the balloon required could also be reduced.

To investigate the sensitivity of the results to changes in the number of balloons and flow exit velocity, calculations are also performed for delivering the same total amount of material using 8 and 40 balloons, and maximum (exit) flow velocities of 10 and 50 m s^−1^. The results are shown in table 3. Varying the number of balloons has relatively little effect on the pumping pressure and travel time at low exit velocities. This is because there is a pressure contribution of 429 kPa due to the static head, which is invariant to changes in mass flow rate of SO_2_, pipe radius and flow velocity and can be considered the minimum required pumping pressure at this temperature. Any additional pumping pressure required is due to the frictional pressure loss contribution, which increases as the exit velocity increases and the pipe radius diminishes. The static head pressure contribution can vary with temperature; as the fluid temperature decreases, the fluid density increases and hence the static head increases. At 450 K, the static head is 160 kPa, and at 550 K, the static head is estimated to be 86.5 kPa (based on extrapolated fluid properties, as reliable experimental data are only available up to 525 K). Unlike hydrogen, where the static head pressure contribution was negligible for the chosen parameter sets, for the case of SO_2_ there is a clear benefit in operating the pumping system at elevated temperatures in order to reduce the minimum required pumping pressure.

**Table 3.** Required pumping pressures for varying numbers of balloons and varying exit velocities.

no. balloons	4			8			40		
mass flow rate SO_2_ (kg s^−1^)	132			66.0			13.2		
exit velocity (m s^−1^)	10	50	100	10	50	100	10	50	100
pipe radius (m)	5.89	2.63	1.86	4.16	1.86	1.32	1.86	0.833	0.589
pumping pressure (kPa)	431	528	865	432	565	993	435	700	1470
travel time (minutes)	597	145	119	597	156	137	601	193	203

### 4.3 Titanium dioxide in a nitrogen-supported slurry

The density of liquid N_2_ is approximately 800 kg m^−3^ at 1 bar, 77 K on the condensation line, thus the static head generated by a 20 km column of liquid N_2_ is about 160 MPa. With additional frictional pressure losses, the required base pressure would exceed both the solidification pressure and the maximum bursting pressure of the pipe, and hence N_2_ must also be pumped in the gas/supercritical fluid phases. Unlike SO_2_, this can be achieved at temperatures at or below ambient.

**Figure 6.**
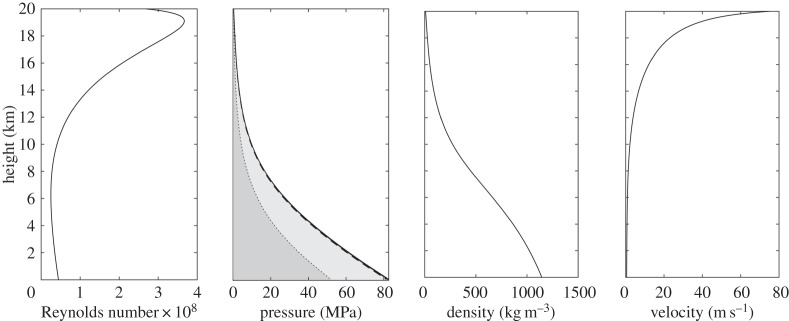
Variation of fluid properties for pumping TiO_2_/N_2_ at 250 K. The pressure contribution from the static head is indicated by the dotted line and the dark shaded region; the contribution from frictional pressure loss is shown by the light shaded region and the change in fluid momentum, shown by the darkest region, is very small. The solid line shows the total pressure requirement.

For this isothermal analysis, a temperature of 250 K will be assumed. As the N_2_ is loaded with TiO_2_ particles (of density 4230 kg m^−3^) that will require dispersion at the top of the pipe, an exit pressure is required, and 300 kPa is estimated to be adequate. Setting the maximum fluid velocity to 100 m s^−1^ and the outlet pressure to 300 kPa, the required radius to give a mass flow rate of 298 kg s^−1^ is 0.395 m. The resulting variation of fluid properties with height is shown in figure 6. The Reynolds number assumes a constant dynamic viscosity of 15.5 μPa s, and the required pumping pressure at ground level is 82.6 MPa. This value is approaching the maximum allowable pressure for Kevlar 49 and PBO.

To investigate the sensitivity of the results to changes in the phase density, number of balloons and flow exit velocity, calculations are also performed for delivering the same total amount of material using phase densities of 0.3 and 0.9, 8 and 40 balloons, and maximum (exit) flow velocities of 10 and 50 m s^−1^. The results are shown in tables 4–6. Similar to the SO_2_ case, it can be seen that varying the number of balloons has little effect on the pumping pressure and travel time for TiO_2_/N_2_. This is again due to the static head pressure contribution, which is 51.9 MPa for a phase density of 0.5, 218 MPa for a phase density of 0.3, and 5.92 MPa for a phase density of 0.9. As the static head pressure contribution for a phase density of 0.3 exceeds the maximum allowable pressure for Kevlar 49 and PBO, it can be concluded that, within the limited isothermal assumption, pumping with this low phase density is infeasible. As for SO_2_, the static head pressure contribution will reduce as the pumping temperature increases, so pumping with lower phase densities will become feasible at elevated temperatures.

**Table 4.** Required pumping pressures for a phase density of 0.5.

no. balloons	4			8			40		
mass flow rate TiO_2_ (kg s^−1^)	99.3			49.7			9.93		
mass flow rate N_2_ (kg s^−1^)	199			99.3			19.9		
exit velocity (m s^−1^)	10	50	100	10	50	100	10	50	100
pipe radius (m)	1.25	0.559	0.395	0.884	0.395	0.280	0.395	0.177	0.125
pumping pressure (MPa)	52.1	61.3	82.6	52.1	64.1	88.4	52.5	73.0	103.5
travel time (minutes)	1450	340	228	1450	355	244	1460	404	285

**Table 5.** Required pumping pressures for a phase density of 0.3.

no. balloons	4			8			40		
mass flow rate TiO_2_ (kg s^−1^)	99.3			49.7			9.93		
mass flow rate N_2_ (kg s^−1^)	331			166			33.1		
exit velocity (m s^−1^)	10	50	100	10	50	100	10	50	100
pipe radius (m)	1.61	0.721	0.511	1.14	0.511	0.361	0.510	0.228	0.161
pumping pressure (MPa)	218	221	230	218	221	233	218	224	243
travel time (minutes)	7020	1420	739	7020	1426	749	7030	1445	782

**Table 6.** Required pumping pressures for a phase density of 0.9.

no. balloons	4			8			40		
mass flow rate TiO_2_ (kg s^−1^)	99.3			49.7			9.93		
mass flow rate N_2_ (kg s^−1^)	110			55.2			11.0		
exit velocity (m s^−1^)	10	50	100	10	50	100	10	50	100
Pipe radius (m)	0.931	0.416	0.294	0.659	0.295	0.208	0.294	0.132	0.093
pumping pressure (MPa)	6.35	18.2	37.4	6.51	21.0	42.5	7.17	29.4	55.8
travel time (minutes)	132	74.3	75.8	136	85.9	85.6	149	120	111

## 5. Limitations of this study

This study has presented an isothermal analysis of the pumping of hydrogen, SO_2_ and a TiO_2_/N_2_ mixture from ground level to 20 km. These isothermal conditions can be considered to be a convenient, but somewhat unrealistic, limiting case. In reality, the fluid temperature will change with time and distance due to a number of heat flow considerations. These include the combined process of conduction through the pipe wall and convection as the wind moves across the pipe; heating of the fluid due to friction between the fluid and the pipe wall; convection within the fluid; the Joule–Thomson effect for real gases; and ambient temperature gradients. The US Standard Atmosphere model defines ambient temperature as 25°C at ground level, decreasing linearly with altitude to −60°C at 11 km, and then −60°C from 11 to 20 km [22].

These heat flows and their effect on the pumping analysis are currently under investigation. The adiabatic assumption, which is analogous to a perfectly insulated tether, provides an alternative limiting case for assessment of the pumping requirements. This type of analysis is beyond the scope of this paper and requires accurate enthalpy data over a range of temperatures and pressures for each of the considered materials. Obtaining such data for the TiO_2_/N_2_ mixture may be problematic. For the SO_2_ case shown in figure 5, an adiabatic analysis results in a required pumping pressure of 274 kPa (cf. 865 kPa using isothermal analysis), and a fluid temperature that decreases from 625 K at ground level to 350 K at 20 km. For the hydrogen cases considered in §4.1, adiabatic analysis indicates a temperature variation of only 10°C along the entire tether, and good agreement with the isothermal prediction for pumping pressure. These two examples illustrate the significance of heat flow in this pumping analysis, and the need for more detailed calculations, to facilitate a better assessment of the feasibility of these pumping systems.

## 6. Conclusion

An initial isothermal pumping analysis of the transport of fluids along a vertical 20 km pipe has shown that the pumping feasibility is highly dependent on the phase characteristic of the material. For the three materials examined in this paper, hydrogen, SO_2_ and titanium dioxide particles suspended in nitrogen, the gas phase is required to avoid solidification. The analysis indicates that, within the range of parameter values considered and the limiting case of isothermal analysis, pumping sufficient hydrogen to power a fuel cell and replenish a balloon can be achieved at temperatures below 0°C. The pumping regime for hydrogen replenishment is dominated by frictional effects, which means that the required pumping pressure decreases as the amount of material to be pumped increases. Pumping of SO_2_ is problematic, as temperatures above 0°C are required to achieve the gas phase, and this will accelerate creep failure in the pipe's strength elements. While the required pumping pressures from this analysis are feasible for SO_2_, the required pipe size results in a heavy system with large drag forces due to wind effects. These drag forces could be reduced by varying the pipe radius along the length of the tether. Pumping of TiO_2_/N_2_ under isothermal conditions requires pressures that are approaching the maximum allowable pipe pressure for the proposed strength fibres. The pumping regime for both the SO_2_ and the TiO_2_/N_2_ cases is dominated by the static head pressure contribution, hence varying the number of balloons has little effect on the required pumping pressures. The static head can be decreased by using elevated temperatures and, for TiO_2_/N_2_, a higher phase density.
